# Udiometrical, Thermometrical, and Barometrical Statements

**Published:** 1809-05

**Authors:** T. Pole

**Affiliations:** Bristol


					Mr. Pole's Table of Temperature, $rc. 435 '
JJdiometricalf Thermometrical, and Barotnetrical Statements,,
by Dr. T. Pole, of Bristol.
An Account of the Quantity of Rain fallen in each Month since
the Year 1802, as ascertained by a correctr Rain Gauge, kept
by Dr. T. Pole, James's Square, Bristol; ? Also, the Average
Tbmperature of each Month since the Year 1803, from Ob-
servations made at Eight o?Glock in the Morning ; r? Also, the
Average State of the Barometer, from Observations made at
the same Hour; ? To which are added, Tables of the Highest
and Lowest Tepiper^tures of the Atmosphere, for the same
Period.
An
Account of the Quantity, of Rain fallen in each Month, since
the \ ear 1802, as ascertained by a correct Rain Gauge.
Months, as de-
nominated in
the Calendar.
1803
o
?
o
1 804
IS05
<1806
1807
c
i w
a> Q
"5 ?
c o
1803
a
C3
>w
o
o
G>
January - -
February - -
March - -
April - - -
May
June --
July - - -
August - -
September -
October - -
November
December
0 48
1
2
3
80
55
15
0 94
1 81
1 56
0 55
3 80
6 19
44
30
98
78
43
58
60
22
59
94
32
73
28
15
34
49
82
15
21
55
69
14
44
5
} *
0 53
0 35
5 37
99
75,
76
6
36
2 6
8
1 52
Total 27 39129 77126 1
34 38(31 31(32 8
Th?
Dr. Pale's Table of Temperature, 3;c.
The average Temperature of each Month since the Year 1S03,
from Observations ma'de at 8 in ihe Morning..
January
February
March
April
May
June
July
August
Septemb.
October
Novemb.
Decemb.
The average State of the Barometer for each Month since the Year
1803.
Months, a? de-
nominate^ in
.the Calendar.
January -
February -
March -
April - t
May - ?
June - r
July - -
August r*
September
October -
November
December
Dr. Pole*$ Table of Temperature, fyc, 437
^hc highest Temperature of the Atmosphere, indicated by the
Therpiometer, at any one Time during the Summers of the last
Five Years.
BJ
rt ^
e a
_ o
*3 e
u
?r ?
? ^
E Z
I ?
6
7
9
6
7
Months, as de-
nominated in
the calendar.
June - -
July - -
September
June - - >|
July - r
July * *
O 3
c/5 ?*
9 fe
? p-
6X)
<U
Q
Years,
86
86
77
84
85
1804
1805
1806
1807
1808
The lowest Temperature of the Atmosphere, indicated by thft
Thermometer, on the two coldest Days in the last Five Years,
according to Observations made at eight o'CJeck in the Morn*
ing.
g S
*5 ?
? "5
5
Months, as de-
nominated in
the calendar.
?Xj
c
c?" S
Q
3
12
2
11
1
l
1.2
1
12
March
December
February -
November
January -
October
January -
December
January -
December
23
24&30|
2
21
30
24
16
23
22
21

				

